# Supplementary material for: Chromothripsis during telomere crisis is independent of NHEJ, and consistent with a replicative origin
Source: Genome Res. 2019 May;29(5):737–49. doi: 10.1101/gr.240705.118 (PMC6499312; doi:10.1101/gr.240705.118)
Supplement: Supplemental Material [file supp_gr.240705.118_Supplemental_file_1.zip › contigs/annotated_contigs/DB79/contig.2.DB79_length_531_mean_cov_3.28060263653.docx]

**DB79_length_531_mean_cov_3.28060263653**

```
CTTTGCCCTTGACCTTACAAACGTGTAGGAACGTTGCCCTCGCTTCAATTCCACAGCTCTGTTTGTTTTCACTCCATAATGGTTTGTTT
 >chr1:180222892-180223206 - E=2e-177
CTCAACTCTTCACAGAGATCCACATGGCAGGTTTCTCCCATGGACCACAGCCCACCCCGTTCTGCCAGGCTAACCCCCCACTAACTGAG

GAGCTCCAGGCAGTTAAGAGTCATCTCCATGGGAAGCTCTCAAGGCTGGTACAGGGGAGACCAGGAAAGCCAGCGCAGGCCCCGCCCTG

AGGGAACTCTGACTACCTGACCCTGCAGGGCTGCGGGTCAAGGGGCC|TATTTTACCTCAATAAAAAATTGTTTAGGAGGAGTAAAAAA
                                                 >chr1:180196470-180196685 - E=1e-111
AATGCCAAAGTGTAAAAGTGGTTATCTGTGCAGTGGATATTTTATTTTATTCTCTTCGCTTTTTTCAGTTTTCTCCATGAGCACATTAT

TTTATAAAGCAGAAAAAAACTAATAAAATGCTTTTTAAAATATTAGGGAAAAGGTAAGGAAAGATACCAAAATCCTATTCAAAAG|CA
```
